# Supplementary material for: The Relationships between Screen Use and Health Indicators among Infants, Toddlers, and Preschoolers: A Meta-Analysis and Systematic Review
Source: Int J Environ Res Public Health. 2020 Oct 7;17(19):7324. doi: 10.3390/ijerph17197324 (PMC7579161; doi:10.3390/ijerph17197324)
Supplement: Supplementary file 1 [file ijerph-17-07324-s001.pdf]

The relationships between screen use and health indicators among infants, toddlers and preschoolers: A meta-analysis and systematic review

Appendix

Supplemental Files 1:

Table S1. The characteristics of the studies included in this review

Table S2. GRADE assessment of quality of evidence reported the associations between media use and health indicators.

Table S1. The characteristics of the studies included in this review

Author, date	Study design	N	Age group (months)	Country	Measurements	The way described screen time use	Media devices examined	Screen time group	Health domain	Health indicators	Quality
Ariza et al. 2004[1]	Cross-sectional study	80	60-72	America (Mexican American)	Maternal report: survey	Viewing categories (hours/day)	TV	≤3<br>>3	Physical	Adiposity (Overweight (BMI:25-29.9))	6
Barr et al. 2008[2]	Quasi-experimental design	120	12(40)<br>15(40)<br>18(40)	America	Parent report: survey	Mean (SD) (hours/day)	TV	3.67(2.46)	Psycho-social<br>Physical	Parent interaction (Responsiveness)<br>Cognitive development (Learning)	Low bias (Selective bias)
Bedford et al. 2016[3]	Cross-sectional study	612	6-36	England	Parent report: survey	Mean (SD) (hours/day)	Touchscreen	0.41(0.65)	Physical	Cognitive development (Language development)<br>Motor development (Fine motor, Gross motor)	7
Beyens et al. 2019[4]	Cross-sectional study	402	36-60	America	Maternal report	Mean (SD) (hours/day)	TV<br>Mobile devices<br>Tablet devices<br>Handheld game player<br>Laptop computer<br>Smartphone	3.8(2.64)<br>3.08(4.08)<br>1.4(2.11)<br>0.59(1.39)<br>0.51(1.33)<br>0.41(1.11)	Behavioral	Sleep problems	6
Brambilla et al 2017[5]	Cross-sectional study	523	0-36	Italy	Parent report:	Prevalence	TV	39.6%	Behavioral	Sleep problems	7

					survey(interview)						
Brockmann et al. 2016[6]	Cross-sectional study	100	12-72	Chile	Parent report: survey	Median (min-max) (hours/day)	TV	0.042(0.008,0.018)	Behavioral	Sleep problems	7
Byeon et al. 2015[7]	Cross-sectional study	1778	24-30	Korea	Parent report: survey	Mean (SD) (hours/day)	TV	1.21(0.99)	Physical	Cognitive development (Language development)	7
Cespedes et al. 2014[8]	Cohort study	1864	12-84	America	Parent report: survey	Mean (SD) (hours/day)	TV	6months:0.9 (1.2)	Behavioral	Sleep problems(duration)	7
Chen et al..2019[9]	Cross-sectional study	714	0-6(321)<br>6-24(393)	Singapore	Parent report: survey	Median (IQR) (hours/day)	ALL<br>TV<br>Mobile devices<br>Computer	1.00 (0.50–2.00)<br>0.98 (0.43–2.00)<br>0.50 (0.25–1.00)<br>0.50 (0.36–1.00)	Behavioral	Sleep problems(duration)	7
Cheng et al. 2010[10]	Cohort study	302	18-30	Japan	Maternal report: survey	Viewing categories (hours/day)	TV	<1<br>≥1 to <3<br>≥3 to <4<br>≥4	Psycho-social (Behavioral and emotional outcomes)	Emotional symptoms<br>Conduct problems<br>Hyperactivity-Inattention<br>Peer-problems<br>Prosocial behaviors	7
Cheung et al.2017[11]	Cross-sectional study	612	6-36	England	Parent report	Mean (SD) (hours/day)	Touchscreen	0.41(0.65)	Behavioral	Sleep duration<br>Sleep latency	5
Christakis et al. 2007[12]	Cohort study	330	24-60	America	Parent report: survey	Mean (SD) (hours/day)	TV (content)	Educational:0.42 (0.60)<br>Nonviolent entertainment:0.91 (1.00)	Psycho-social	Aggressive behavior (Antisocial behaviors)	6

								Violent entertainment:0.54 (0.80)			
Collings et al. 2018[13]	Cohort study	1338	12; 18; 24(798); 36(909)	England	Parent report: survey	Mean (SD) (hours/day)	TV	All time points:1.3(1.2)<br>36 months:2.1(1.5)<br>24 months:1.3(2)	Physical	Adiposity (BMI, Sum of skinfolds, Waist circumference)	7
Cox et al. 2012[14]	Cross-sectional study	135	24-72	Australia	Maternal report: survey	Mean (SD) (hours/day)	TV	Weekday:1.43(0.92)<br>Weekend:1.72(1.11)	Physical<br>Behavioral	Adiposity (BMI z-score)<br>Healthy dietary behavior (Energy intake, fast foods, vegetables)	7
De Coen et al..2014[15]	cohort study	568	36-72	Belgium	Parent report: survey	Viewing categories (hours/day)	ALL	Weekday:>1; ≤1<br>Weekend:>2; ≤2	Physical	Adiposity (Overweight)	7
Ebenegger et al. 2012[16]	Cross-sectional study	450	48-72	Swiss	Parent report: survey	Mean (SD) (hours/day)	TV	0.93(0.82)	Psycho-social	Behavioral and emotional outcomes (Hyperactivity/Inattention)	7
Foster et al. 2010[17]	Cohort study	1159	12, 36	America	Parent report	Viewing categories (hours/day)	TV	0<br>0-1<br>1-2<br>2-3<br>3-4<br>4-5<br>5-6<br>6-7<br>≥7	Psycho-social	Behavioral and emotional outcomes (Attentional problems)	7
Garrison et al. 2011[18]	RCT	617	36-60	America	Parent report:	Mean (SD) (hours/day)	TV	Daytime: 0.98(0.71)<br>Evening:0.24(0.29)	Behavioral	Sleep problems	Low bias (Measur

					survey(interview)						ement bias)
Garrison et al. 2012[19]	RCT	565	36-60	America	Parent report: survey(interview)	Content	TV	Unhealthy media: violent or age-inappropriate content	Behavioral	Sleep problems	Low bias (Measurement bias)
Gubbels et al. 2009[20]	Cross-sectional study	2471	24	Netherlands	Parent report: survey	Viewing categories (hours/day)	TV<br>Computer	0<br>0.01-0.24<br>0.25-0.99<br>1-1.99<br><2<br>≥2	Behavioral	Healthy dietary behavior quality (Snacks, Brown bread, Fresh fruit, Sugar-sweetened drinks, Light soft drinks, Vegetables, White bread)	7
Helm et al. 2019[21]	Cross-sectional study	470	33-71	America	Parent report: survey	Viewing categories (hours/day)	TV	0<br><1<br>1-3<br>4-6<br>≥ 7	Behavioral	Sleep problems	8
Hinkley et al. 2017[22]	Cohort study	108	36-60	Australia	Parent report: survey	Mean (SD) (hours/day)	TV<br>Sedentary electronic games<br>Active electronic games<br>Computer/ internet (excluding games)	1.4(0.85)<br>0.12(0.29)<br>0.04(0.09)<br>0.10(0.23)	Psycho-social	Emotional and social skills (Intrapersonal, Stress management, Interpersonal, Adaptability)	7
Hinkley et al. 2014[23]	Cohort study	3604	24-72	Belgium; Cyprus;		Mean (SD) (hours/day)	Weekday television	Boy:1.04 (0.75); Girl:0.98(0.70)	Psycho-social	Behavioral and emotional outcomes	8

				Estonia; Germany; Hungary; Italy; Spain; Sweden	Parent report: survey		Weekend television<br>Weekday PC<br>Weekend PC	Boy:1.62 (0.93); Girl: 1.53 (0.91)<br>Boy:0.19 (0.39); Girl:0.11 (0.26)<br>Boy:0.33 (0.56); Girl:0.21 (0.40)		(Emotional problems, Peer problems, Self-esteem, Emotional well-being)	
Howie et al. 2017[24]	Subject laboratory trial	6	36-60	Australia	Parent report	Range (hours/day)	mobile touchscreen	(0.33,2.7)	Behavioral<br>Physical	Sedentary behaviors<br>Musculoskeletal risk	Low bias (Selective bias)
Hu et al. 2019[25]	Case-control study	933	12-24(264)<br>48-60(669)	China	Parent report: survey(interview)	Viewing categories (hours/day)	TV	<1<br>≥1	Physical<br>Behavioral	Adiposity (Overweight)<br>Sleep problems) Sleep duration<br>Physical activity	7
Inoue et L. 2016[26]	Cohort study	32439	36-60	Japan	Parent report: survey	Viewing categories (hours/day)	TV	0<br><1<br>1-2<br>2-3<br>3-4<br>4-5<br>>5	Psycho-social	Emotional and social skills (Self-Regulation)	7
Jago et al. 2005[27]	Cohort study	149<br>147<br>133	36-48<br>48-60<br>60-72	America	Maternal report	not mentioned	TV	not mentioned	Physical	Adiposity (BMI)	7
Ji et al. 2018[28]	Cross-sectional study	112	36-72	China	Parent report: survey	Mean (SD) (hours/day)	ALL	Weekday:0.75(0.7); Weekend:2.07(1.64)	Behavioral	Sleep problems	7
Jouret et al. 2007[29]		298(boy)	48	France	Parent report	not mentioned	TV	not mentioned	Physical	Adiposity (Overweight (BMI≥90% percent))	7

	Cross-sectional study	295(girl)									
Kirkorian et al. 2016[30]	Cross-sectional study	58(boy)<br>58(girl)	23.5-36	America	Parent report: survey	Range (hours/day)	ALL<br>TV<br>Interactive media	(0,3.58)<br>(0,3.33)<br>(0,1)	Physical	Cognitive development (Language learning)	6
Lillard et al. 2011[31]	Cross-sectional study	60	48	America	Parent report: survey	Range (hours/day)	Fast-Paced Television<br>Educational Television<br>Drawing	0.8(0.16)<br>0.66(0.16)<br>0.91(0.16)	Physical	Executive function	6
Lin et al. 2015[32]	Cross-sectional study	150	15-36	China	Parent report: survey(interview)	Mean (hours/day)	TV	2.28	Physical	Cognitive development<br>Cognitive development (Language development)<br>Motor development	7
Lumeng et al. 2006[33]	Cross-sectional study	1016	36	America	Maternal report	Viewing categories(hours/day)	TV	<2<br>≥2	Physical	Adiposity (Overweight (BMI≥95%))	7
Manganello et al. 2009[34]	Cohort study	3128	36	America	Maternal report	Mean (SD) (hours/day)	TV	3.2(2.3)	Psycho-social	Aggressive behavior	7
Manios et al. 2009[35]	Cohort study	2241	12-69	Greece	Parental report	Mean (SD) (hours/day)	TV	1.32(1.12)	Physical	Adiposity (Overweight(95%>BMI≥85%), Obese (BMI≥95%))	7
Marinell et al. 2014[36]	Cohort study	1444	24(657)<br>48(787)	Spain	Maternal report	Viewing categories(hours/day)	TV	<0.5<br>≥0.5	Behavioral	Sleep duration	7
		185	43.2-57.6	Australia			ALL	2.42(1.33)	Physical	Executive Function	6

McNeill et al. 2019[37]	Cohort study				Parent report: survey	Mean (SD) (hours/day)	TV	2.04(1.14)	Psycho-social	Behavioral and emotional outcomes (Internalizing problems, Externalizing problems)	
Mendoza et al. 2007[38]	Cross-sectional study	1809	24-60	America	Parent report: survey	Viewing categories(hours/day)	ALL	>2	Physical	Adiposity (Overweight, Sum of skinfolds)	7
								≤2			
							TV	>2			
								≤2			
							Computer	0			
								<1			
								1			
								2			
								3			
								4			
								≥5			
Mezie-Okoye et al. 2015[39]	Cross-sectional study	198	24-60	Nigeria	Maternal report	Viewing categories(hours/day)	ALL	≤1	Physical	Adiposity (Overweight (BMI≥85%))	8
								>1			
Miller et al. 2007[40]	Cohort study	107	34.8-70.4	America	Parent report: survey(interview)	not mentioned	TV	not mentioned	Physical	Behavioral and emotional outcomes (Attentional problems)	6
Nathanson et al. 2014[41]	Cross-sectional study	107	38-74	America	Parent report: survey	Mean (SD) (hours/day)	TV (background)	2.87(1.99)	Physical	Executive function	6
Nathanson et al. 2018[42]	cross-sectional study	402	36-60	America	Maternal report	Mean (SD) (hours/day)	TV	3.8(2.64)	Behavioral	Sleep problems (Sleep duration, Sleep latency)	7
							Tablet devices	1.4(2.11)			
							Smartphone	0.41(1.11)			
							Game player	0.59(1.39)			

							Laptop computer	0.51(1.33)			
							iPod	0.17(0.78)			
Nevarez et al. 2010[43]	Cohort study	1676<br>1228<br>1365	6<br>12<br>24	America	Maternal report	Mean (SD) (hours/day)	TV	6months:0.89 (1.21)<br>12months:1.20 (1.46)<br>24months:1.44 (1.22)	Behavioral	Sleep problems(duration)	7
Paavonen et al. 2006[44]	Cross-sectional study	321	60-72	Finland	Parent report: survey	Viewing categories(hours/day)	TV (content)	<1.66<br>≥1.66	Behavioral	Sleep problems	7
Pagani et al. 2013[45]	Cohort study	1997	29	Canada	Parent report: survey	Mean (SD) (hours/day)	TV	29months: 1.62(1.21)	Psycho-social	Bullying	
		1997	65						Physical	Cognitive development (mathematics, learning)<br>Motor development (Gross motor)	7
Parent et al. 2016[46]	Cohort study	209	36-84	America	Parent report: survey	Viewing categories(hours/day)	ALL	0-2<br>2.1-4<br>4.1-6<br>6.1-8<br>8.1-10<br>>10	Behavioral	Behavioral problems<br>Sleep problems	6
Parkes et al. 2013[47]	Cohort study	11014<br>11014	60<br>84	England	Parent report	Viewing categories(hours/day)	Television/video/DVD<br>Computer/electronic games	0<br><1<br>≥1,<3<br>≥3,<5<br>≥5,<7	Psycho-social	Behavioral and emotional outcomes (Conduct problems, Hyperactivity/inattention, Emotional symptoms,	7

								≥7		Peer problems, Prosocial behavior)	
Peck et al. 2015[48]	Cross-sectional study	12385	48-72	America	Parent report: survey	Viewing categories(hours/day)	TV	≥1;<1<br>≥2;<2	Physical	Adiposity (Obesity, Overweight)	7
Pinhas-Hamiel et al. 2009[49]	Cross-sectional study	204	48-72	Israeli	Parent report: survey	Mean (SD) (hours/day)	ALL	Normal weight (122):4(1.8)<br>Normal weight (51):4(2.2)<br>Normal weight (31):4.6(2.5)	Physical	Adiposity (Overweight)	7
Plancoulaine et al. 2018[50]	Cohort study	1205	60-72	France	Maternal report	Mean (SD) (hours/day)	TV	0.76(0.7)	Behavioral	Sleep duration	7
Raman et al. 2017[51]	Cross-sectional study	210	12-36	America	Parent report: survey	not mentioned	ALL	not mentioned	Psycho-social	Social-emotional delay	7
Reilly et al. 2005[52]	Cohort study	8234	0-36	England	Parent report: survey	not mentioned	TV	not mentioned	Physical	Adiposity (Obesity)	7
Ribner et al. 2017[53]	RCT	807	60.88-76.84	America	Parent report	Mean (SD) (hours/day)	TV	2.19(0.79)	Psycho-social	Cognitive development (language development, mathematics)	no bias
									Physical	Executive function	
Ruangdaraganon et al. 2009[54]	Cohort study	220	0-24	Thailand	Parent report: survey(interview)	Mean (SD) (hours/day)	TV	12months:1.23(1.42)<br>24months:1.69(1.56)	Physical	Cognitive development (language development)	8
		759	36-48				ALL	1.02(0.67)	Physical	Adiposity (BMI)	7

Sijtsma et al. 2015[55]	Cohort study			Netherlands	Parent report: survey	Mean (SD) (hours/day)	TV<br>Computer	0.97(0.63)<br>0.05(0.15)	Behavioral	Sleep problems (Sleep duration)	
Sisson et al. 2012(a)[56]	Cross-sectional study	1368	24-60	America	Parent report: survey	Viewing categories(hours/day)	TV	≤1<br>2-3<br>≥4	Behavioral	Healthy dietary behavior (Energy intake)	6
Sisson et al. 2012(b)[57]	Cross-sectional study	1423	24-60	America	Parent report: survey	Viewing categories(hours/day)	TV	≤1<br>2-3<br>≥4	Behavioral	Healthy dietary behavior (Dietary quality)	6
Skaug et al. 2018[58]	Cohort study	995<br>795	48<br>72	Norway	Parent report: survey(interview)	Mean (SD) (hours/day)	TV	0.92(0.57)<br>1.1(0.87)	Psycho-social	Parent interaction (Responsiveness)	7
Sorrie et al..2016[59]	cross-sectional study	504	36-60	Ethiopia	Caregiver report: survey(interview)	Viewing categories(hours/day)	TV	≤2<br>>2	Physical	Adiposity (Overweight)	6
Straker et al. 2006[60]	Cohort study	1600	60	Australia	Parent report: survey	Viewing categories(hours/day)	TV<br>Hand-held games<br>Console games	0<br><1<br>1-2<br>>2	Physical	Musculoskeletal risk	8
Sugimori et al. 2004[61]	Cohort study	8170<br>8170	36<br>72	Japan	Parent report: survey	not mentioned	TV	not mentioned	Physical	Adiposity (Overweight)	7
Suglia et al. 2013[62]	Cohort study	1589<br>1589	36<br>60	America	Maternal report	Viewing categories(hours/day)	TV	≤2<br>>2	Physical	Adiposity (Obesity)	6
Tahir et al. 2019[63]	Cohort study	30921	36-60	America	Maternal report: survey	Viewing categories(hours/day)	TV	0<br>0.5<br>1<br>2	Physical	Adiposity (Overweight)	7

								3<br>≥4			
Tamana et al. 2019[64]	Cohort study	2427<br>2427	36<br>60	Canada	Parent report	Mean (95%CI) (hours/day)	ALL	1.8(1.5,2.1)	Psycho-social (Behavioral and emotional outcomes)	Externalizing behaviors problems<br>Internalizing behaviors problems	7
Taveras et al. 2006[65]	Cross-sectional study	240	24-70.8	America	Parent report	Mean (SD) (hours/day)	TV	1.4(1.1)	Behavioral	Healthy dietary behavior quality (Fast Food Intake)	7
Tester et al. 2018[66]	Cohort study	7028	24-60	America	Parent report: survey	Viewing categories(hours/day)	TV<br>ALL	>2<br>>3<br>>2<br>>3<br>>4	Physical	Adiposity (Overweight (BMI:85%-94.5%), Obesity (BMI:95%-1.2*95%), Severe obesity (BMI≥1.2*95%))	5
Thompson et al. 2005[67]	cross-sectional study	1783	4-35	America	Parent report: survey (telephone interview)	Mean (95%CI) (hours/day)	TV	4-11months:0.9 (0.8–1.0)<br>12-23months:1.6 (1.4–1.8)<br>24-35months:2.3 (2.1–2.5)	Behavioral	Sleep problems (Naptime/Bedtime Schedules)	7
Tomopoulos et al. 2010[68]	Cohort study	259	6	America	Maternal report: survey(interview)	Mean (SD) (hours/day)	ALL	2.55(2.09)	Physical	Cognitive development<br>Cognitive development (language development)	7
		75	21	America			ALL	21months: 1.97(1.8)		Aggressive behavior	7

Tomopoulos et al. 2007[69]	Cohort study	96	33		Caregiver report: survey(interview)	Mean (SD) (hours/day)		33months: 2.13(1.59)	Psycho-social	Behavioral and emotional outcomes (Oppositional Defiant problems, Externalizing problems, Hyperactivity problems)	
Tso et al. 2016[70]	Cross-sectional study	533	65.5	China	Parent report: survey	Viewing categories(hours/day)	ALL	≤1<br>>1-3<br>≥3	Behavioral	Sleep problems (Sleep duration)	7
Twenge et al. 2019[71]	Cross-sectional study	13109	0-12(3818)<br>24-60(9291)	America	Parent report: survey	Viewing categories(hours/day)	TV and Video game<br>Portable electronic devices	<2<br>≥2	Behavioral	Sleep problems (Sleep duration)	7
Vallejo et al. 2015[72]	Cross-sectional study	140	24-72	Brazil	Maternal report	not mentioned	TV	not mentioned	Physical	Adiposity (Overweight)	6
Verlinden et al. 2014[73]	Cohort study	1176	24-60	Netherlands	Parent report: survey	Viewing categories	TV	Low<br>Mid-low<br>Mid-high<br>High	Psycho-social	Bullying (Being a bully, Being a bully-victim, Being a victim)	6
Vijakkhana et al. 2015[74]	Cohort study	208<br>208	6<br>12	Thailand	Caregiver report	Mean (SD) (hours/day)	ALL	6 months:4.46(2.98)<br>12months:4.81(3.03)	Behavioral	Sleep problems (Sleep duration)	7
Watt et al. 2015[75]	Cohort study	1997	29	Canada	Maternal report	Mean (SD) (hours/day)	TV	1.26(0.88)	Psycho-social	Bullying (Victimization at 12 years old)	7
Wu et al. 2017[76]		8900	36-72	China	Caregiver report		ALL	<2	Psycho-social	Behavioral and emotional outcomes	6

	Cross-sectional study					Viewing categories(hours/ day)		≥2		(Emotional symptoms, Conduct problems, Hyperactivity, Peer problems, Prosocial)	
Xu et al..2016[77]	RCT	497<br>415<br>369	24<br>42<br>60	Australia	Maternal report	Mean (SD) (hours/day)	ALL	1.37 (1.02)<br>2.48 (1.49)<br>2.25 (1.27)	Behavioral	Sleep problems (Sleep duration)<br>Sleep problems (Sleep latency)	no bias
Zhao et al. 2018[78]	Cross-sectional study	18562	36-48	China	Parent report: survey	Viewing categories(hours/ day)	ALL	0-1<br>1-2<br>2-3<br>3-4<br>>4	Psycho-social	psychosocial problems	8
Zimmerman et al. 2005[79]	Cohort study	1797<br>1797	0-35<br>36-60	America	Maternal report	Mean (SD) (hours/day)	TV	0-35months: 2.2(2.22)<br>36-60months: 3.29(2.18)	Physical	Cognitive development (mathematics, reading comprehension. reading recognition)	7
Zimmerman et al. 2007[80]	Cross-sectional study	729	8-16(384)<br>17-24(345)	America	Parent report: survey (telephone interview)	Mean (SD) (hours/day)	DVDs/videos	8-16 months: 0.15(0.35)<br>17-24 months: 0.16(0.43)	Physical	Cognitive development (language development)	5
							Children's educational shows	8-16 months: 0.31(0.7)<br>17-24 months: 0.7(0.96)			
							Movies and children's non-educational TV	8-16 months: 0.16(0.49)<br>17-24 months: 0.39(0.75)			

	Grownup TV	8-16 months: 0.1(0.31) 17-24 months: 0.15(0.41)	

Note:

RCT: randomized control trail

Table S2. GRADE assessment of quality of evidence reported the associations between media use and health indicators.

No. of participants (No. of studies)	Design	Risk of bias	Quality assessment				Summary of findings	
			Inconsistency	Indirectness	Imprecision	Publication bias	Absolute effect	Quality of evidence (GRADE)
Adiposity								
Observational studies								
933 (1)[25]	Case-control study	No serious limitation	No serious inconsistency	No serious indirectness	No serious imprecision	Undetected	TV (duration)<br>1/1 studies reported positive associations	Low
60997(10)[13, 15, 27, 35, 52, 55, 61-63, 66]	Cohort study	No serious limitation	No serious inconsistency	No serious indirectness	No serious imprecision	Undetected	TV (duration)<br>8/8 studies reported positive associations<br>Screen time (duration)<br>2/2 studies reported positive associations	High
17064 (10)[1, 14, 29, 33, 38, 39, 48, 49, 59, 72]	Cross-sectional study	No serious limitation	No serious inconsistency	No serious indirectness	No serious imprecision	Undetected	TV (duration)<br>7/7studies reported positive associations<br>Screen time (duration)<br>2/3 studies reported positive associations<br>1/3 studies reported negative associations	Moderate
Cognitive development								
Intervention studies								
807 (1)[53]	RCT	No serious limitation	No serious inconsistency	No serious indirectness	No serious imprecision	Undetected	TV (duration)<br>1/1 study reported null associations	Moderate
Observational studies								

4273 (4)[45, 54, 68, 79]	Cohort study	No serious limitation	No serious inconsistency	No serious indirectness	No serious imprecision	Undetected	TV (duration)<br>2/3studies reported negative associations<br>1/3 studies reported null associations<br>Screen time (duration)<br>1/1 studies reported negative associations	Low
3385 (5)[3, 7, 30, 32, 80]	Cross-sectional study	No serious limitation	No serious inconsistency	No serious indirectness	No serious imprecision	Undetected	TV (duration)<br>2/2 studies reported negative associations<br>Touchscreen (duration)<br>1/1 studies reported null associations<br>DVD/videos (duration)<br>1/1 studies reported null associations<br>Screen time (duration)<br>1/1 studies reported positive associations	Low
Executive function								
Intervention studies								
805 (1)[53]	RCT	No serious limitation	No serious inconsistency	No serious indirectness	No serious imprecision	Undetected	TV (duration)<br>1/1 studies reported negative associations	Moderate
Observational studies								
185 (1)[37]	Cohort study	No serious limitation	No serious inconsistency	No serious indirectness	No serious imprecision	Undetected	Screen time (duration)<br>1/1 studies reported negative associations	Low
167 (2)[31, 41]	Cross-sectional study	No serious limitation	No serious inconsistency	No serious indirectness	No serious imprecision	Undetected	TV (duration)<br>2/2 studies reported negative associations	Low
Motor development								
Observational studies								
1997 (1)[45]	Cohort study	No serious limitation	No serious inconsistency	No serious indirectness	No serious imprecision	Undetected	TV (duration)<br>1/1 studies reported negative associations	Low
762 (2)[3, 32]	Cross-sectional study	No serious limitation	No serious inconsistency	No serious indirectness	No serious imprecision	Undetected	TV (duration)<br>1/1 studies reported negative associations	Low

							Touchscreen (duration)<br>1/1 studies reported mixed negative and null associations	
Musculoskeletal risk								
Intervention studies								
6 (1)[24]	Subject laboratory trial	Serious limitation	No serious inconsistency	No serious indirectness	No serious imprecision	Undetected	Touchscreen (duration)<br>1/1 studies reported positive associations	Moderate
Observational studies								
1600 (1)[60]	Cohort study	No serious limitation	No serious inconsistency	No serious indirectness	No serious imprecision	Undetected	TV (duration)<br>1/1 studies reported positive associations	Low
Healthy dietary behavior quality								
Observational studies								
5637 (5)[14, 20, 56, 57, 65]	Cross-sectional study	No serious limitation	No serious inconsistency	No serious indirectness	No serious imprecision	Undetected	TV (duration)<br>5/5 studies reported negative associations	Low
Physical activity								
Observational studies								
933 (1)[25]	Case-control study	No serious limitation	No serious inconsistency	No serious indirectness	No serious imprecision	Undetected	TV (duration)<br>1/1 studies reported negative associations	Low
Sedentary activity								
Intervention studies								
6 (1)[24]	Subject laboratory trial	Serious limitation	No serious inconsistency	No serious indirectness	No serious imprecision	Undetected	Touchscreen (duration)<br>1/1 studies reported positive associations	Moderate
Sleep problems								
Intervention studies								
1679 (3)[18, 19, 77]	RCT	No serious limitation	No serious inconsistency	No serious indirectness	No serious imprecision	Undetected	TV (duration)<br>2/2 studies reported positive associations<br>Screen time (duration)<br>1/1 studies reported positive associations	Moderate
Observational studies								

933 (1)[25]	Case-control study	No serious limitation	No serious inconsistency	No serious indirectness	No serious imprecision	Undetected	TV (duration)<br>1/1 studies reported positive associations	Low
7365 (7)[8, 36, 43, 46, 50, 55, 74]	Cohort study	No serious limitation	No serious inconsistency	No serious indirectness	No serious imprecision	Undetected	TV (duration)<br>5/5 studies reported positive associations<br>Screen time (duration)<br>2/2 studies reported positive associations	Moderate
19081 (12)[4-6, 9, 11, 21, 28, 42, 44, 67, 70, 71]	Cross-sectional study	No serious limitation	No serious inconsistency	No serious indirectness	No serious imprecision	Undetected	TV (duration)<br>5/6 studies reported positive associations<br>1/6 studies reported negative associations<br>TV (prevalence)<br>1/1 studies reported positive associations<br>Touchscreen (duration)<br>1/1 studies reported positive associations<br>Video games (duration)<br>1/1 studies reported positive associations<br>Screen time (duration)<br>3/3 studies reported positive associations	Moderate
Aggressive behavior<br>Observational studies								
3533 (3)[11, 34, 69]	Cohort study	No serious limitation	No serious inconsistency	No serious indirectness	No serious imprecision	Undetected	TV (duration)<br>2/2 studies reported positive associations<br>Screen time (duration)<br>1/1 studies reported positive associations	Low
Behavioral and emotional outcomes<br>Observational studies								
19103 (9)[10, 17, 23, 37, 40, 46, 47, 64, 69]	Cohort study	No serious limitation	No serious inconsistency	No serious indirectness	No serious imprecision	Undetected	TV (duration)<br>5/5 studies reported negative associations<br>Computer games<br>1/1 studies reported negative associations<br>Screen time (duration)	Moderate

							4/4 studies reported negative associations	
28122 (4)[16, 76]	Cross-sectional study	No serious limitation	No serious inconsistency	No serious indirectness	No serious imprecision	Undetected	TV (duration)<br>1/1 studies reported negative associations<br>Screen time (duration)<br>2/2 studies reported negative associations<br>Touchscreen(duration)<br>1/1 studies reported mixed negative, positive and null associations	Moderate
Bullying								
Observational studies								
5170 (3)[73, 75]	Cohort study	No serious limitation	No serious inconsistency	No serious indirectness	No serious imprecision	Undetected	TV (duration)<br>3/3 studies reported positive associations	Low
Social and emotional skills								
Observational studies								
32547 (2)[22, 26]	Cohort study	No serious limitation	No serious inconsistency	No serious indirectness	No serious imprecision	Undetected	TV (duration)<br>1/2 studies reported positive associations<br>1/2 studies reported negative associations	Low
Parental interaction (Responsiveness)								
Intervention studies								
120 (1)[2]	Quasi-experimental design	Serious limitation	No serious inconsistency	No serious indirectness	No serious imprecision	Undetected	TV (duration)<br>1/1 studies reported positive associations	Moderate
Observational studies								
995 (1)[58]	Cohort study	No serious limitation	No serious inconsistency	No serious indirectness	No serious imprecision	Undetected	TV (duration)<br>1/1 studies reported positive associations	Low

Note: GRADE Working Group grades of evidence.

High quality: Further research is very unlike to change our confidence in the estimate of effect.
Moderate quality: Further research is likely to have an impact on our confidence in the estimate of effect and may change the estimate.
Low quality: Further research is very likely to influence our confidence in the estimate of effect and is likely to change the estimate.
Very low quality: Uncertain about the estimate.

Reference

1. Ariza AJ, Chen EH, Binns HJ, Christoffel KK. Risk Factors for Overweight in Five- to Six-Year-Old Hispanic-American Children: A Pilot Study. Journal of Urban Health. 2004;81(1):150-61.
2. Barr R, Zack E, Garcia A, Muentener P. Infants' attention and responsiveness to television increases with prior exposure and parental interaction. Infancy. 2008;13(1):30-56.
3. Bedford R, de Urabain IRS, Cheung CHM, Karmiloff-Smith A, Smith TJ. Toddlers' Fine Motor Milestone Achievement Is Associated with Early Touchscreen Scrolling. Frontiers in Psychology. 2016;7.
4. Beyens I, Nathanson AI. Electronic Media Use and Sleep Among Preschoolers: Evidence for Time-Shifted and Less Consolidated Sleep. Health Communication. 2019;34(5):537-44.
5. Brambilla P, Giussani M, Pasinato A, Venturelli L, Privitera F, Miraglia Del Giudice E, et al. Sleep habits and pattern in 1-14 years old children and relationship with video devices use and evening and night child activities. Italian journal of pediatrics. 2017;43(1):7.
6. Brockmann PE, Diaz B, Damiani F, Villarroel L, Núñez F, Bruni O. Impact of television on the quality of sleep in preschool children. Sleep Medicine. 2016;20:140-4.
7. Byeon H, Hong S. Relationship between television viewing and language delay in toddlers: Evidence from a Korea national cross-sectional survey. PLoS ONE. 2015;10(3).
8. Cespedes EM, Gillman MW, Kleinman K, Rifas-Shiman SL, Redline S, Taveras EM. Television viewing, bedroom television, and sleep duration from infancy to mid-childhood. Pediatrics. 2014;133(5):e1163-e71.
9. Chen B, van Dam RM, Tan CS, Chua HL, Wong PG, Bernard JY, et al. Screen viewing behavior and sleep duration among children aged 2 and below. BMC public health. 2019;19(1):59.
10. Cheng S, Maeda T, Yoichi S, Yamagata Z, Tomiwa K. Early television exposure and children's behavioral and social outcomes at age 30 months. Journal of epidemiology / Japan Epidemiological Association. 2010;20 Suppl 2:S482-9.
11. Cheung CHM, Bedford R, De Urabain IRS, Karmiloff-Smith A, Smith TJ. Daily touchscreen use in infants and toddlers is associated with reduced sleep and delayed sleep onset. Scientific Reports. 2017;7.
12. Christakis DA, Zimmerman FJ. Violent television viewing during preschool is associated with antisocial behavior during school age. Pediatrics. 2007;120(5):993-9.
13. Collings PJ, Kelly B, West J, Wright J. Associations of TV Viewing Duration, Meals and Snacks Eaten When Watching TV, and a TV in the Bedroom with Child Adiposity. Obesity. 2018;26(10):1619-28.
14. Cox R, Skouteris H, Rutherford L, Fuller-Tyszkiewicz M, Dell' Aquila D, Hardy LL. Television viewing, television content, food intake, physical activity and body mass index: a cross-sectional study of preschool children aged 2-6 years. Health promotion journal of Australia : official journal of Australian Association of Health Promotion Professionals. 2012;23(1):58-62.
15. De Coen V, De Bourdeaudhuij I, Verbestel V, Maes L, Vereecken C. Risk factors for childhood overweight: a 30-month longitudinal study of 3- to 6-year-old children. Public health nutrition. 2014;17(9):1993-2000.
16. Ebenegger V, Marques-Vidal PM, Munsch S, Quartier V, Nydegger A, Barral J, et al. Relationship of hyperactivity/inattention with adiposity and lifestyle characteristics in preschool children. Journal of Child Neurology. 2012;27(7):852-8.
17. Foster EM, Watkins S. The value of reanalysis: TV viewing and attention problems. Child Development. 2010;81(1):368-75.
18. Garrison MM, Christakis D. The sustained impact of a healthy media use intervention on preschool

child sleep.
19. Garrison MM, Christakis DA. The Impact of a Healthy Media Use Intervention on Sleep in Preschool Children. Pediatrics. 2012;130(3):492-9.
20. Gubbels JS, Kremers SPJ, Stafleu A, Dagnelie PC, de Vries SI, de Vries NK, et al. Clustering of Dietary Intake and Sedentary Behavior in 2-Year-Old Children. Journal of Pediatrics. 2009;155(2):194-8.
21. Helm AF, Spencer RMC. Television use and its effects on sleep in early childhood. Sleep health. 2019;5(3):241-7.
22. Hinkley T, Timperio A, Salmon J, Hesketh K. Does Preschool Physical Activity and Electronic Media Use Predict Later Social and Emotional Skills at 6 to 8 Years? A Cohort Study. Journal of physical activity & health. 2017;14(4):308-16.
23. Hinkley T, Verbestel V, Ahrens W, Lissner L, Molnar D, Moreno LA, et al. Early Childhood Electronic Media Use as a Predictor of Poorer Well-being A Prospective Cohort Study. Jama Pediatrics. 2014;168(5):485-92.
24. Howie EK, Coenen P, Campbell AC, Ranelli S, Straker LM. Head, trunk and arm posture amplitude and variation, muscle activity, sedentariness and physical activity of 3 to 5 year-old children during tablet computer use compared to television watching and toy play. Applied Ergonomics. 2017;65:41-50.
25. Hu JJ, Ding N, Yang L, Ma YA, Gao M, Wen DL. Association between television viewing and early childhood overweight and obesity: a pair-matched case-control study in China. Bmc Pediatrics. 2019;19.
26. Inoue S, Yorifuji T, Kato T, Sanada S, Doi H, Kawachi I. Children's Media Use and Self-Regulation Behavior: Longitudinal Associations in a Nationwide Japanese Study. Maternal and child health journal. 2016;20(10):2084-99.
27. Jago R, Baranowski T, Baranowski JC, Thompson D, Greaves KA. BMI from 3-6y of age is predicted by TV viewing and physical activity, not diet. International Journal of Obesity. 2005;29(6):557-64.
28. Ji MM, Tang A, Zhang YF, Zou JJ, Zhou GY, Deng J, et al. The Relationship between Obesity, Sleep and Physical Activity in Chinese Preschool Children. International Journal of Environmental Research and Public Health. 2018;15(3).
29. Jouret B, Ahluwalia N, Cristini C, Dupuy M, Nègre-Pages L, Grandjean H, et al. Factors associated with overweight in preschool-age children in southwestern France. American Journal of Clinical Nutrition. 2007;85(6):1643-9.
30. Kirkorian HL, Choi K, Pempek TA. Toddlers' Word Learning From Contingent and Noncontingent Video on Touch Screens. Child development. 2016;87(2):405-13.
31. Lillard AS, Peterson J. The immediate impact of different types of television on young children's executive function. Pediatrics. 2011;128(4):644-9.
32. Lin LY, Cherng RJ, Chen YJ, Chen YJ, Yang HM. Effects of television exposure on developmental skills among young children. Infant Behavior and Development. 2015;38:20-6.
33. Lumeng JC, Rahnama S, Appugliese D, Kaciroti N, Bradley RH. Television exposure and overweight risk in preschoolers. Archives of Pediatrics and Adolescent Medicine. 2006;160(4):417-22.
34. Manganello JA, Taylor CA. Television Exposure as a Risk Factor for Aggressive Behavior Among 3-Year-Old Children. Archives of Pediatrics & Adolescent Medicine. 2009;163(11):1037-45.
35. Manios Y, Kourlaba G, Kondaki K, Grammatikaki E, Anastasiadou A, Roma-Giannikou E. Obesity and television watching in preschoolers in greece: The genesis study. Obesity. 2009;17(11):2047-53.
36. Marinelli M, Sunyer J, Alvarez-Pedrerol M, Iñiguez C, Torrent M, Vioque J, et al. Hours of television viewing and sleep duration in children a multicenter birth cohort study. JAMA Pediatrics. 2014;168(5):458-64.

37. McNeill J, Howard SJ, Vella SA, Cliff DP. Longitudinal Associations of Electronic Application Use and Media Program Viewing with Cognitive and Psychosocial Development in Preschoolers. Academic Pediatrics. 2019;19(5):520-8.
38. Mendoza JA, Zimmerman FJ, Christakis DA. Television viewing, computer use, obesity, and adiposity in US preschool children. International Journal of Behavioral Nutrition and Physical Activity. 2007;4.
39. Mezie-Okoye MM, Alex-Hart B. Overweight and obesity among preschool children in Port Harcourt, Nigeria. Pakistan Journal of Nutrition. 2015;14(4):209-13.
40. Miller CJ, Marks DJ, Miller SR, Berwid OG, Kera EC, Santra A, et al. Brief report: Television viewing and risk for attention problems in preschool children. Journal of Pediatric Psychology. 2007;32(4):448-52.
41. Nathanson AI, Aladé F, Sharp ML, Rasmussen EE, Christy K. The relation between television exposure and executive function among preschoolers. Developmental psychology. 2014;50(5):1497-506.
42. Nathanson AI, Beyens I. The Relation Between Use of Mobile Electronic Devices and Bedtime Resistance, Sleep Duration, and Daytime Sleepiness Among Preschoolers. Behavioral sleep medicine. 2018;16(2):202-19.
43. Nevarez MD, Rifas-Shiman SL, Kleinman KP, Gillman MW, Taveras EM. Associations of early life risk factors with infant sleep duration. Academic Pediatrics. 2010;10(3):187-93.
44. Paavonen EJ, Pennonen M, Roine M, Valkonen S, Lahikainen AR. TV exposure associated with sleep disturbances in 5-to 6-year-old children. Journal of Sleep Research. 2006;15(2):154-61.
45. Pagani LS, Fitzpatrick C, Barnett TA. Early childhood television viewing and kindergarten entry readiness. Pediatric research. 2013;74(3):350-5.
46. Parent J, Sanders W, Forehand R. Youth Screen Time and Behavioral Health Problems: The Role of Sleep Duration and Disturbances. Journal of developmental and behavioral pediatrics : JDBP. 2016;37(4):277-84.
47. Parkes A, Sweeting H, Wight D, Henderson M. Do television and electronic games predict children's psychosocial adjustment? Longitudinal research using the UK Millennium Cohort Study. Archives of Disease in Childhood. 2013;98(5):341-8.
48. Peck T, Scharf RJ, Conaway MR, DeBoer MD. Viewing as little as 1 hour of TV daily is associated with higher change in BMI between kindergarten and first grade. Obesity. 2015;23(8):1680-6.
49. Pinhas-hamiel O, Bar-zvi E, Boyko V, Reichman B, Lerner-geva L. Prevalence of overweight in kindergarten children in the centre of Israel - Association with lifestyle habits. Child: Care, Health and Development. 2009;35(2):147-52.
50. Plancoulaine S, Reynaud E, Forhan A, Lioret S, Heude B, Charles MA, et al. Night sleep duration trajectories and associated factors among preschool children from the EDEN cohort. Sleep Medicine. 2018;48:194-201.
51. Raman S, Guerrero-Duby S, McCullough JL, Brown M, Ostrowski-Delahanty S, Langkamp D, et al. Screen exposre during daily routines and a young child's risk for having social-emotional delay. Clinical Pediatrics. 2017;56(13):1244-53.
52. Reilly JJ, Armstrong J, Dorosty AR, Emmett PM, Ness A, Rogers I, et al. Early life risk factors for obesity in childhood: cohort study. Bmj-British Medical Journal. 2005;330(7504):1357-9.
53. Ribner A, Fitzpatrick C, Blair C. Family Socioeconomic Status Moderates Associations Between Television Viewing and School Readiness Skills. Journal of developmental and behavioral pediatrics : JDBP. 2017;38(3):233-9.

54. Ruangdaraganon N, Chuthapisith J, Mo-suwan L, Kriweradechachai S, Udomsubpayakul U, Choprapawon C. Television viewing in Thai infants and toddlers: Impacts to language development and parental perceptions. BMC Pediatrics. 2009;9.
55. Sijtsma A, Koller M, Sauer PJ, Corpeleijn E. Television, sleep, outdoor play and BMI in young children: the GECKO Drenthe cohort. European journal of pediatrics. 2015;174(5):631-9.
56. Sisson SB, Broyles ST, Robledo C, Boeckman L, Leyva M. Television viewing and variations in energy intake in adults and children in the USA. Public health nutrition. 2012;15(4):609-17.
57. Sisson SB, Shay CM, Broyles ST, Leyva M. Television-viewing time and dietary quality among U.S. children and adults. American Journal of Preventive Medicine. 2012;43(2):196-200.
58. Skaug S, Englund KT, Wichstrøm L. Young children's television viewing and the quality of their interactions with parents: A prospective community study. Scandinavian journal of psychology. 2018;59(5):503-10.
59. Sorrie MB, Yesuf ME, Gebremichael TG. Overweight/Obesity and associated factors among preschool children in Gondar City, Northwest Ethiopia: A cross-sectional study. PLoS ONE. 2017;12(8).
60. Straker LM, Pollock CM, Zubrick SR, Kurinczuk JJ. The association between information and communication technology exposure and physical activity, musculoskeletal and visual symptoms and socio-economic status in 5-year-olds. Child: Care, Health and Development. 2006;32(3):343-51.
61. Sugimori H, Yoshida K, Izuno T, Miyakawa M, Suka M, Sekine M, et al. Analysis of factors that influence body mass index from ages 3 to 6 years: A study based on the Toyama cohort study. Pediatrics International. 2004;46(3):302-10.
62. Suglia SF, Duarte CS, Chambers EC, Boynton-Jarrett R. Social and behavioral risk factors for obesity in early childhood.
63. Tahir MJ, Willett W, Forman MR. The Association of Television Viewing in Childhood With Overweight and Obesity Throughout the Life Course. American Journal of Epidemiology. 2019;188(2):282-93.
64. Tamana SK, Ezeugwu V, Chikuma J, Lefebvre DL, Azad MB, Moraes TJ, et al. Screen-time is associated with inattention problems in preschoolers: Results from the CHILD birth cohort study. Plos One. 2019;14(4).
65. Taveras EM, Sandora TJ, Shih MC, Ross-Degnan D, Goldmann DA, Gillman MW. The association of television and video viewing with fast food intake by preschool-age children. Obesity. 2006;14(11):2034-41.
66. Tester JM, Phan TT, Tucker JM, Leung CW, Dreyer Gillette ML, Sweeney BR, et al. Characteristics of Children 2 to 5 Years of Age With Severe Obesity. Pediatrics. 2018;141(3).
67. Thompson DA, Christakis DA. The association between television viewing and irregular sleep schedules among children less than 3 years of age. Pediatrics. 2005;116(4):851-6.
68. Tomopoulos S, Dreyer BP, Berkule S, Fierman AH, Brockmeyer C, Mendelsohn AL. Infant media exposure and toddler development. Archives of Pediatrics and Adolescent Medicine. 2010;164(12):1105-11.
69. Tomopoulos S, Dreyer BP, Valdez P, Flynn V, Foley G, Berkule SB, et al. Media Content and Externalizing Behaviors in Latino Toddlers. Ambulatory Pediatrics. 2007;7(3):232-8.
70. Tso W, Rao N, Jiang F, Li AM, Lee SL, Ho FKW, et al. Sleep Duration and School Readiness of Chinese Preschool Children. Journal of Pediatrics. 2016;169:266-71.
71. Twenge JM, Hisler GC, Krizan Z. Associations between screen time and sleep duration are primarily driven by portable electronic devices: evidence from a population-based study of U.S. children ages 0–

17. Sleep Medicine. 2019;56:211-8.
72. Vallejo M, Cortes-Rodríguez BA, Colin-Ramirez E. Maternal Underestimation of Child's Weight Status and Health Behaviors as Risk Factors for Overweight in Children. Journal of pediatric nursing. 2015;30(6):e29-e33.
73. Verlinden M, Tiemeier H, Veenstra R, Mieloo CL, Jansen W, Jaddoe VWV, et al. Television viewing through ages 2-5 years and bullying involvement in early elementary school. Bmc Public Health. 2014;14.
74. Vijakkhana N, Wilaisakditipakorn T, Ruedeekhajorn K, Pruksananonda C, Chonchaiya W. Evening media exposure reduces night-time sleep. Acta Paediatrica, International Journal of Paediatrics. 2015;104(3):306-12.
75. Watt E, Fitzpatrick C, Derevensky JL, Pagani LS. Too Much Television? Prospective Associations Between Early Childhood Televiewing and Later Self-reports of Victimization by Sixth Grade Classmates. Journal of developmental and behavioral pediatrics : JDBP. 2015;36(6):426-33.
76. Wu X, Tao S, Rutayisire E, Chen Y, Huang K, Tao F. The relationship between screen time, nighttime sleep duration, and behavioural problems in preschool children in China. European Child and Adolescent Psychiatry. 2017;26(5):541-8.
77. Xu H, Wen LM, Hardy LL, Rissel C. Associations of outdoor play and screen time with nocturnal sleep duration and pattern among young children. Acta paediatrica (Oslo, Norway : 1992). 2016;105(3):297-303.
78. Zhao J, Zhang Y, Jiang F, Ip P, Ho FKW, Zhang Y, et al. Excessive Screen Time and Psychosocial Well-Being: The Mediating Role of Body Mass Index, Sleep Duration, and Parent-Child Interaction. Journal of Pediatrics. 2018;202:157-62.e1.
79. Zimmerman FJ, Christakis DA. Children's television viewing and cognitive outcomes: A longitudinal analysis of national data. Archives of Pediatrics and Adolescent Medicine. 2005;159(7):619-25.
80. Zimmerman FJ, Christakis DA, Meltzoff AN. Associations between Media Viewing and Language Development in Children Under Age 2 Years. Journal of Pediatrics. 2007;151(4):364-8.
